# COVID‐19 plasma proteome reveals novel temporal and cell‐specific signatures for disease severity and high‐precision disease management

**DOI:** 10.1111/jcmm.17622

**Published:** 2022-12-19

**Authors:** Cristiana Iosef, Claudio M. Martin, Marat Slessarev, Carolina Gillio‐Meina, Gediminas Cepinskas, Victor K. M. Han, Douglas D. Fraser

**Affiliations:** ^1^ Children's Health research Institute London Ontario Canada; ^2^ Lawson Health Research Institute London Ontario Canada; ^3^ Department of Medicine Western University London Ontario Canada; ^4^ Department of Medical Biophysics Western University London Ontario Canada; ^5^ Department of Pediatrics Western University London Ontario Canada; ^6^ Department of Physiology & Pharmacology Western University London Ontario Canada; ^7^ Department of Clinical Neurological Sciences Western University London Ontario Canada

**Keywords:** COVID‐19, disease severity, precision medicine, proteome

## Abstract

Coronavirus disease 2019 (COVID‐19) is a systemic inflammatory condition with high mortality that may benefit from personalized medicine and high‐precision approaches. COVID‐19 patient plasma was analysed with targeted proteomics of 1161 proteins. Patients were monitored from Days 1 to 10 of their intensive care unit (ICU) stay. Age‐ and gender‐matched COVID‐19‐negative sepsis ICU patients and healthy subjects were examined as controls. Proteomic data were resolved using both cell‐specific annotation and deep‐analysis for functional enrichment. COVID‐19 caused extensive remodelling of the plasma microenvironment associated with a relative immunosuppressive milieu between ICU Days 3–7, and characterized by extensive organ damage. COVID‐19 resulted in (1) reduced antigen presentation and B/T‐cell function, (2) increased repurposed neutrophils and M1‐type macrophages, (3) relatively immature or disrupted endothelia and fibroblasts with a defined secretome, and (4) reactive myeloid lines. Extracellular matrix changes identified in COVID‐19 plasma could represent impaired immune cell homing and programmed cell death. The major functional modules disrupted in COVID‐19 were exaggerated in patients with fatal outcome. Taken together, these findings provide systems‐level insight into the mechanisms of COVID‐19 inflammation and identify potential prognostic biomarkers. Therapeutic strategies could be tailored to the immune response of severely ill patients.

## 1 INTRODUCTION

Coronavirus disease 2019 (COVID‐19) is caused by SARS‐CoV‐2 and is associated with greater than one million fatalities world‐wide.^1^, ^2^, ^3^ The disease varies significantly from asymptomatic to severe illness—the latter is associated with organ dysfunction and risk of death. The pathophysiology of severe disease is dominated by dysfunctional immunity, with either intensified immune or low immune responses.^4^, ^5^, ^6^, ^7^ The uncertainty in disease severity and progression makes uniform COVID‐19 management difficult, indicating that patients could benefit from a precision medicine approach.^8^, ^9^, ^10^, ^11^, ^12^ Among the next generation technologies used to investigate COVID‐19,^10^, ^13^, ^14^, ^15^, ^16^, ^17^, ^18^, ^19^, ^20^, ^21^, ^22^, ^23^, ^24^, ^25^ this study is unique as we dissect the cytokine storm and the organ damage profiles.

In this study, we investigated the plasma proteome (1161 proteins) from COVID‐19 patients admitted to the intensive care unit (ICU), and compared with age‐ and sex‐matched COVID‐19‐negative sepsis control patients (SCTR) and healthy control subjects (HCTR). Plasma protein expression was then used to analyse crucial sets of immune mediators to identify novel molecular mechanisms with the following aims: (1) to identify the protein signatures associated with COVID‐19 severity and death, as well as tissue recovery patterns; (2) to map these signatures to specific cell types and functions within clinical phenotypes^23^; and (3) to screen current drugs that can be repurposed for potential therapy.

Our results indicate the presence of a distinct COVID‐19 plasma profile that drives disease severity and identifies plasma biomarkers to aid high‐precision/personalized therapeutics. We found that COVID‐19 induces cellular changes that resulted in (1) enzymatic extracellular remodelling; (2) the release of collagen which activates platelets and induces coagulation; (3) deficient T‐ and B‐cell homing; (4) activation and expression of exhaustion markers on T cells; (5) increased cell death; and (6) deficient regeneration and growth factor signalling in critically damaged organs.

## 2 MATERIALS AND METHODS

### 2.1 Clinical assessment strategies

This study was approved by Western University, Human Research Ethics Board (REB ID# 1670; issued March 20, 2020). Given the unprecedented pandemic situation and the restricted hospital access for substitute decision makers, waived consent was approved based on the Society for Critical Care Medicine statement on ‘Waiver of Informed Consent in Emergency Situations’ (https://www.sccm.org/Communications/Critical‐Connections/Archives/2018/Waiver‐of‐Informed‐Consent‐in‐Emergency‐Situations). With the exception of the healthy control subjects (HCTR), study participants were admitted to our level‐3 academic ICUs at London Health Sciences Centre (London, Ontario) and were suspected of having COVID‐19 based on standard hospital screening procedures. Blood sampling began on ICU admission, Days 3, 7 and 10. COVID‐19 status was confirmed by standard hospital testing of two SARS‐CoV‐2 viral genes using polymerase chain reaction. Patient baseline characteristics were recorded on admission. Multiple Organ Dysfunction Score (MODS) and Sequential Organ Failure Assessment (SOFA) Score were calculated for both COVID‐19 and COVID‐19‐negative patient groups to enable objective comparison of their illness severity. Both patient groups were characterized as having confirmed or suspected sepsis diagnosis using Sepsis 3.0 criteria. Final participant groups were constructed by age‐ and gender‐matching COVID‐19+ patients with COVID‐19 patients and HCTR (Translational Research Centre, London, Ontario); Directed by Dr. D.D. Fraser. All samples were processed rapidly and equally, and plasma was isolated, aliquoted and frozen at −80°C.

### 2.2 Targeted proteomics

Proximity Extension Assay (PEA) was used to measure plasma protein expression and included immune‐recognition with dNTP‐labelled antibodies, extension mediated by polymerases, amplification and detection as described by Lundberg et al.^26^ A control was used to estimate precision (coefficient of variation), a negative control (buffer) used to set background levels and calculate limit of detection (LOD), a plate control (plasma pool) to correct levels between plates, and a reference plasma control to estimate CV between runs. The relative protein quantification is presented as a Normalized Protein Expression (NPX) on a log2 scale. Data generation of NPX consists of normalization to the extension control (known standard), log2‐transformation and level adjustment using the plate control. The PEA was outsourced to OLINK laboratories (Olink Proteomics, Boston, MA).

### 2.3 Bioinformatic analyses

#### 2.3.1 Functional annotation

Two platforms were used for this task: Database for Annotation, Visualization and Integrated Discovery (DAVID: https://david.ncifcrf.gov), and Gene Set Enrichment Analysis (GSEA) software v7.4 updated April 2021, EC San Diego, Broad Institute. GSEA: https://www.gsea‐msigdb.org). Differentially expressed gene‐set comparing groups were filtered to include enriched genes, pathways and biological processes. Enriched Gene Ontology (GO) terms classified by biological processes were identified using either false discovery rate (FDR) algorithms. Significantly enriched Kyoto encyclopedia of genes and genomes pathways were similarly identified. Bonferroni correction was applied to adjust for multiple comparisons. Adjusted *p* value < 0.05 was considered significant. Pathways incompletely described in GSEA were re‐assessed by using PANTHER‐16.0 (12.01.2020‐version) GO and classification system (http://pantherdb.org) supported by the National Human Genome Research Institute and the National Science Foundation (USA). Clinical annotations were provided by our pathology team. Interaction networks and functional enrichment analysis was performed by using STRING DB platform (https://string‐db.org/). Immune‐cell deconvolution was completed by CIBERSORT analysis (https://cibersort.stanford.edu) an analytical tool provided by Stanford University (‘Stanford’) through Dr. A. Newman laboratories who authored this software. CIBERSORT imputed mRNA profiles and provided an estimation of the abundances of candidate‐cell types in a mixed cell population, using gene expression data. Unsupervised hierarchical clustering was performed by either Pearson correlation or Euclidean distance algorithms along with complete linkage to correlate patterns of gene sets (correlation value was colour‐coded, where blue denotes a correlation coefficient of −1.0 and red indicates a Pearson correlation coefficient of 1.0, in the healthy, sepsis and COVID‐19 microenvironments. Immune cell type and signature scores were calculated from mRNA expression as predefined linear combinations (weighted averages) of biologically relevant gene sets. All this work was possible by using the Morpheus software, a tool developed at the Broad Institute (MA, USA) (https://software.broadinstitute.org/morpheus). Other statistical analyses. NCSS (http://www.ncss.com) a statistics package produced and distributed by NCSS, LLC, was used for statistical validation of the online platforms, and graphic representation of the heatmaps and dendrograms. Bar graphs summarize data analysed using by Mann–Whitney U‐test for unpaired data (two‐sided). *p < 0.05; ***p < 0.0001 (GraphPad Prism, version 9). Several public R‐studio packages from Bioconductor were used for the initial global data analysis, as follows: data load (DBI, odbc); data manipulation (tidyr); data visualization (ggplot2, rgl, leaflet); data modelling (tidymodels); data report (shiny); spatial data (maps); web work (xml, github); and R writing (devtools, curl).

#### 2.3.2 Marker validation

Levels of NCF2 biomarker in plasma samples were quantitatively measured by RayBio Custom enzyme‐linked immunosorbent assay (ELISA) Kit (Norcross), while CAS3 and MMP9 markers were measured with Thermo Fisher Scientific ELISA kits (Waltham). All other proteins were measured with antibody microarray (RayBio® L‐Series Antibody Array; Norcross). The manufacturer's protocols were followed for both ELISA and antibody microarray techniques.

## 3 RESULTS

### 3.1 Patient demographics/clinical data

We investigated and compared three age‐ and gender‐matched groups of patients: patients admitted to the ICU with COVID‐19, SCTRs and HCTRs. COVID‐19 patients were investigated for 10 days with plasma sample collected at Days 1, 3, 7 and 10 postadmission.^15^, ^27^, ^28^ Baseline demographic characteristics, comorbidities, laboratory values and chest X‐ray results are reported in Table S1. The COVID‐19 patients developed bilateral pneumonia more frequently, while COVID‐19‐negative patients were more likely to suffer unilateral pneumonia. The mortality rate was 50% for COVID‐19 patients at the time of study.

### 3.2 The COVID‐19 proteome is unique

**FIGURE 1:**
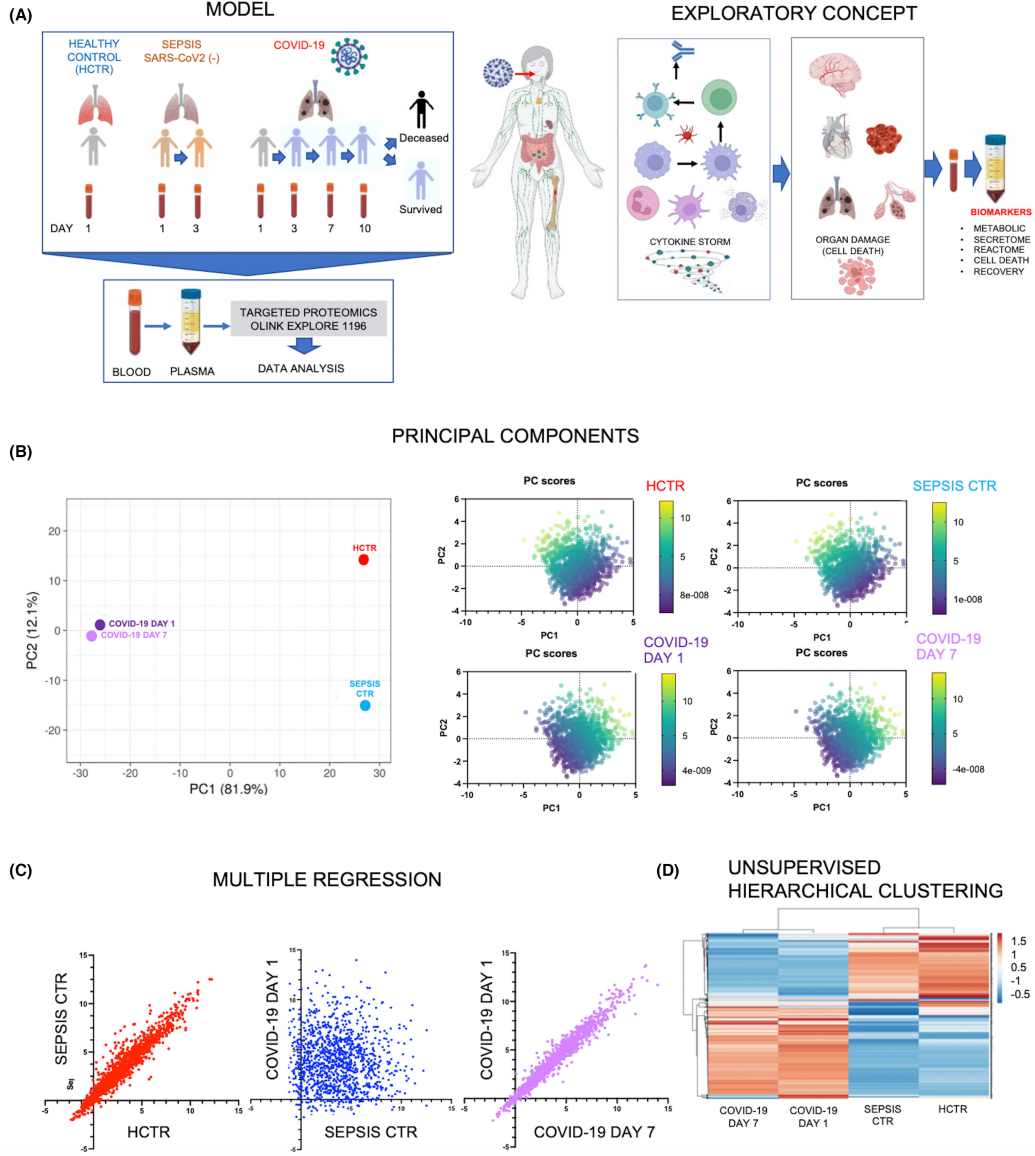
Plasma from COVID‐19 ICU patients has a distinct proteomic profile compared to age‐ and sex‐matched COVID‐19‐negative ICU sepsis patients or healthy control subjects. (A left) Experimental model comprises 30 subjects of which 10 are healthy controls (HCTR, N = 10), the other 10 with COVID‐19‐negative sepsis (SCTR, N = 10) and last 10 with documented COVID‐19 (left panel, study model). (A right) Bulk proteomic data (provided by Olink Explore 1196 panels) have been partitioned as follows: (1) clinical condition (HCTR, SCTR compared to COVID‐19), (2) survival records, Survived and Deceased, (3) timeline (Days 1–10 post admission), (4) ‘secretome storm’ dissection criteria (Interleukins, Chemokines, Growth Factors), (5) cell types and (6) organ damage criteria. (B left) Principal component (PC) analysis (Bioconductor/github//Clustvis tool). Reduced dimensionality of data shows the highest variation in a proportion of 81.9% (PC1) and second most variable in proportion of 12.9% (PC2). High PC1 variability clustered the COVID‐19 data (Days 1 and 7) together at the opposed pole from the other two cohorts. The HCTR and SCTR cohorts clustered separately. These trends were validated by PC score calculation (B right) using NCSS software, multiple‐variable function. (C) Multiple regression analysis (NCSS) shows that HCTR and SCTR data points are highly correlated (*r* > 90%). Similarly, COVID‐19 Days 1 and 7 showed high correlation (*r* > 92%). However, the SCTR (Day 1) versus COVID‐19 (Day 1) groups indicate minimal correlation (*r* < 0.015). (D) Unsupervised hierarchical clustering (by NCSS, 2021) reinforces the unicity of the COVID‐19 profile compared to HCTR and SCTR groups [Z score = (−0.5) to 1.5]

Plasma proteomic profiles from COVID‐19 patients were compared to SCTR patients and HCTR subjects using proximal extension assay (Figure 1A). Principal components were created to determine the data variation with PC1 containing the most variation (81.9%) and PC2 as the second (12.1%) (Figure 1B). The principal components analysis (PCA) plot showed clusters based on their similarity, where HCTR and SCTR proteomes were distinct from COVID‐19 proteomes (ICU Days 1 and 7). Additional plots reinforced the above observations; each PC had one dimension, and the mid‐point had value 0.^26^, ^27^ Furthermore, multiple regression (Figure 1C) indicated high correlation with *R* > 95% for the cross analysis of the control groups (HCTR vs. SCTR) and COVID‐19 Day 1 versus Day 7, but not for the COVID‐19 groups when plotted against either control. Finally, hierarchical clustering algorithms grouped the COVID‐19 proteomes closely together, as opposed to the controls (Figure 1D).

### 3.3 Cell‐type deconvolution indicates neutrophil reprogramming and lymphocyte exhaustion

**FIGURE 2:**
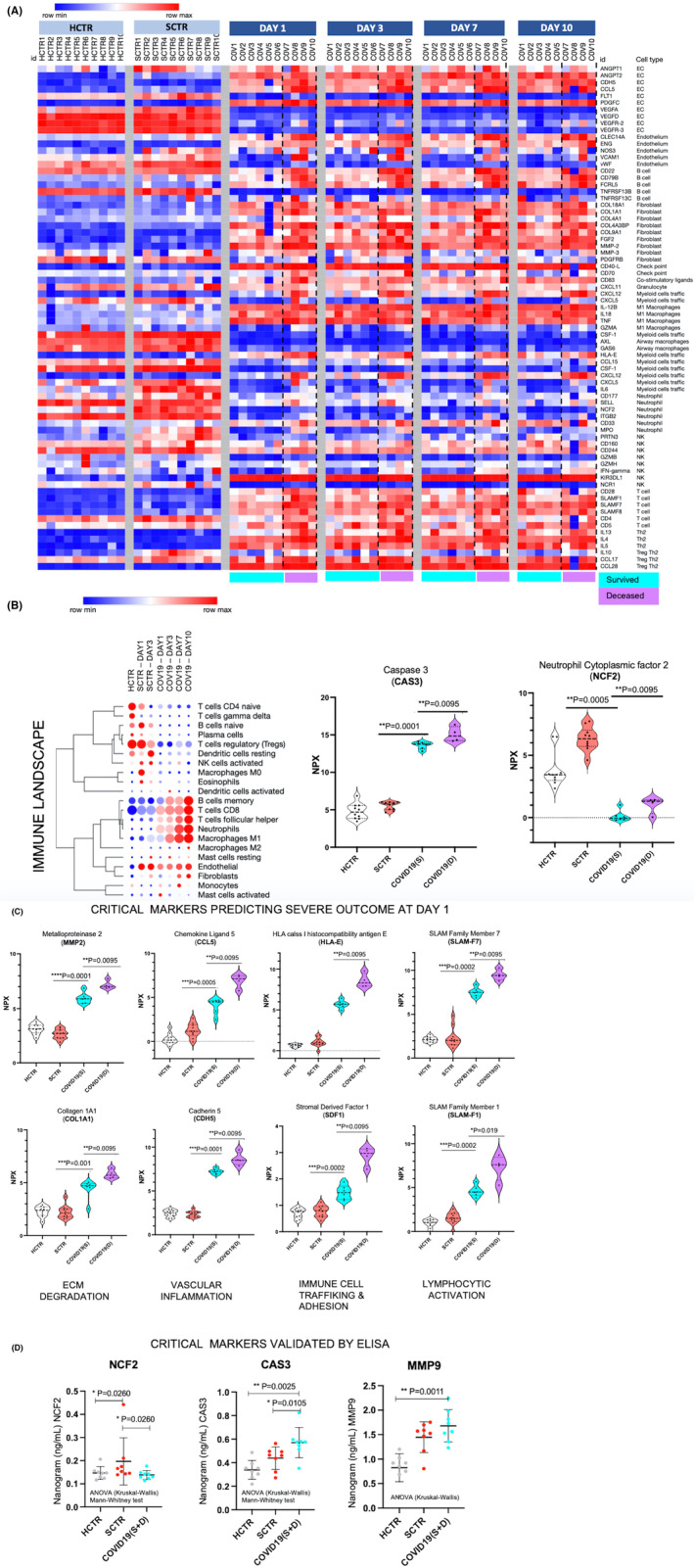
Temporal and cell‐type‐specific proteomic landscape of COVID‐19 plasma indicates defective extracellular matrix as a potential trigger for vascular inflammation and limited homing of the T/B cells. (A) Heatmaps show curated canonic marker signatures^32^ denoting cell types. Canonic sets were used to analyse bulk data resulted from Olink Explore 1196 panels. Markers associated with each cell type are displayed to the right side of the heatmap. Data were temporally partitioned based on the time point when plasma samples were procured. Data visualization (heatmap) was performed using the Morpheus' platform tools repository; Normalized Protein Expression (NPX) variations are denoted on a colour scale where in red are data points above the row‐median (M) and in blue, bellow M. Unsupervised hierarchical clustering was performed using Pearson correlation algorithms built into Morpheus platform. (B left) Bubble map shows the quantitative (%) contribution of different immune cell types to the plasma proteomic profile. Proportions were calculated by using the CIBERSORT engine with the LM22 immune cell‐canonic data set as reference, while endothelial and fibroblasts/stromal cell proportions were calculated by using the EPIC engine (with ‘in tissue’ data set as reference). Data can be visualized based on a colour scale where, red indicates data points above the row M and blue, data below M. The bubble size denotes a potential comparison between data point‐proportions taken per column. Heatmap was obtain with Morpheus system resources as well. (B right) Violin plots demonstrate elevated Caspase 3 (CAS‐3, a lysosomal enzyme involved in the apoptotic pathway) and depressed neutrophil factor 2 (NCF2; necessary for reactive oxygen species (ROS) formation). (C) Critical, representative biomarkers derived from the cellular landscaping denote extracellular matrix (ECM) degradation (MMP2, COL1A1), vascular inflammation (CDH5, CCL5), immune cell trafficking and poor adhesion (SDF1, HLA‐E), T‐cell activation (SLAM1, 7). Violin plots statistical parameters: GraphPad 9, Column analysis, Mann–Whitney non‐parametric t‐test where *p* < 0.05, N = 10 for each group except the COVID‐19‐survived (S) group, where N = 6 and COVID‐19‐deceased group (D) where N = 4. (D) NCF2, MMP9 and CAS3 markers were measured by ELISA, demonstrating similar findings to Olink protein expression methods.

Biomarker signatures associated with specific cell types are presented in Figure 2A, where heatmaps indicate multiple COVID‐19 trends compared with the HCTR and SCTR, as follows: (1) increased endothelial cell (EC) and endothelium associated markers (ANGPT1/2, CCL5, CLEC14A, ENG, NOS3 and CDH5, but not VEGF); (2) B‐lymphocytes represented by CD79B, CD22 and FCRL5, but not TNFRSF13; (3) circulating fibrocytes based on collagen type I, FGF2 and MMP2; (4) downregulated myeloid traffic to included neutrophils depleted of NCF2 (Figure 2B; reprogramming of neutrophils into a predicted phenotype with low reactive oxygen species); (5) NK cell activity downregulated, with increased T‐cell representation (CD4/5 markers and SLAM receptors); and (6) ‘lymphocyte exhaustion’ indicated by of Th2 profile.^29^, ^30^ Significant down‐regulation of Axl and GAS6, two airway macrophage markers, was also observed in COVID‐19.

The CIBERSORT platform^31^ was used to analyse cell populations based on curated cell signatures.^32^ In proportionality, cell populations highly represented in COVID‐19 were CD8+ T cells (associated with high expression of CAS‐3, a cell death indicator), macrophage type 1, neutrophils (with low NCF2 expression), T‐cell follicular helper, B cells, monocytes, ECs and fibrocytes (Figure 2B).

Eight different markers were selected to represent the pathological signatures indicated by the cell type deconvolution (Figure 2C), as follows: MMP2 and COL1A1 (ECM degradation); CCL5 and CDH5 (micro‐vascular inflammation and destabilization); HLA‐E and SDF1 (immune cell trafficking, homing and adhesion); and SLAM‐F1 and SLAMF‐7 (lymphocyte activity). COVID‐19 resulted in elevation of all markers, with higher levels in COVID‐19 fatalities.

Taken together, CD8+ T cells were increased, with signatures that indicated exhaustion (upregulated PD‐1 and IL‐15; downregulated IL‐7), likely secondary to high viral load. Exhausted T cells are considered dysfunctional, typically failing to clear infected cells and with progressive loss of IL‐2, TNFα, IFNγ and granzyme B.^29^, ^30^ Immune checkpoint receptors were upregulated in COVID‐19 (i.e. PD‐1), indicating ‘lymphocyte exhaustion’, as shown previously.^29^, ^30^

### 3.4 ECM phenotypes in COVID‐19 indicate remodelling with limited potential for immune cell homing

**FIGURE 3:**
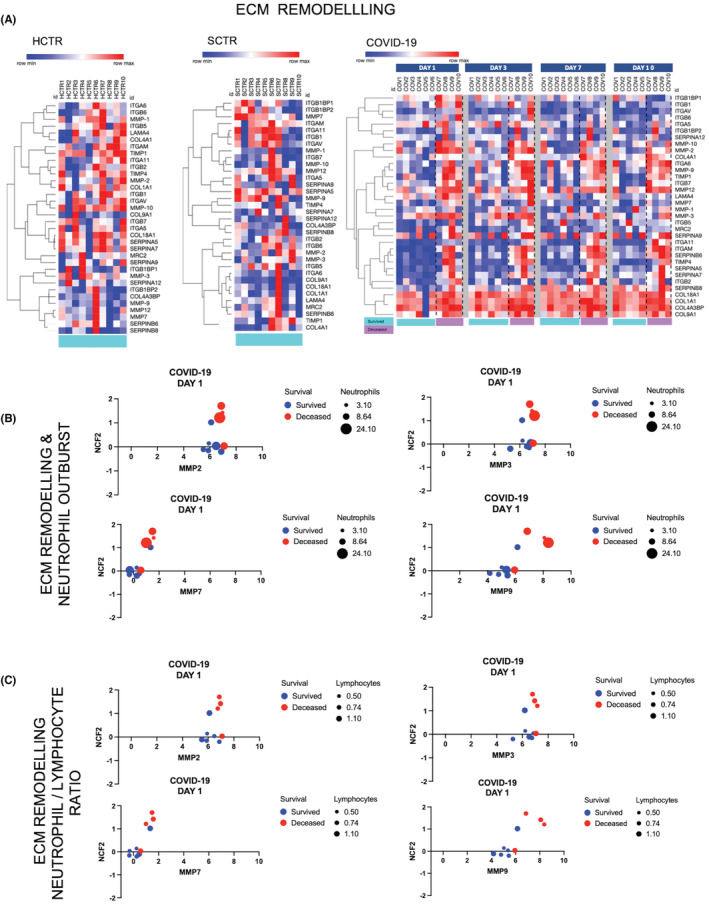
Plasma proteome informs the extracellular matrix (ECM) remodelling process, which evolves with high enzymatic input, thus releasing critical ECM components such as collagen. (A) Heatmaps were obtained using the same machine learning technology described in Figure 2A. Control groups (SCTR and HCTR) were plotted separately because, if taken together in a single map, the minimum and the maximum data point values were exceptionally different for some of the critical biomarkers and the red versus blue colour scale would have misinformed on the biomarker expression. The ECM signature markers included structural components such as collagen subtypes (COL), integrins (ITG) and laminin (LAMA), along with proteases such as metalloproteinases (MMPs) and proteases inhibitors such as serpins and/or TIMP1‐4. MRC1/2 (CD206) a collagen non‐signalling recycling receptor was also selected for the ECM signature markers set. (B, C) Metalloproteinases MMP‐2, MMP‐3, MMP‐7 and MMP‐9 were analysed in a multiple‐variable graph format (bubble graph) in correlation with (1) survival rates (colour code: red = deceased, blue = survived), (2) number of neutrophils or lymphocytes quantified by a bubble size scale, and (3) levels of NCF2 (neutrophil cytoplasmic factor 2). The analysis indicates whether ECM remodelling by MMPs is related to the neutrophil function and if such correlation predicts the disease severity and the fatal outcome in COVID‐19 patients. Lymphocyte numbers were substituted for neutrophils to determine the role of neutrophil/lymphocyte ratio, a known measurement modified in COVID‐19. Correlation was established by Pearson‐based algorithms.

ECM remodelling was detected in COVID‐19 with upregulation of MMP‐2, MMP‐3 and MMP‐9, as well as SERPIN‐A9 and SERPIN‐B6 (Figure 3A). Collagen was released into plasma, together with integrins (ITGA11 and ITGAM), and in the same context, the MRC2/CD206 collagen recycling non‐signalling receptor was reduced. A critical downstream effect could be platelet activation by collagen type I. Furthermore, increased TIMP‐1 and TIMP‐4, two nonspecific MMP inhibitors, were observed in the COVID‐19 samples. This latter observation could reflect compensation for the high release of MMPs, but it could also have implications in cell differentiation, migration and cell death. Indeed, TIMPs activate cellular signalling cascades via CD63, which could function as a blood platelet activation marker, in addition to collagen. As reprograming of neutrophils could contribute to ECM fluctuations in COVID‐19, we explored this hypothesis by correlating MMP‐2, MMP‐3, MMP‐7 and MMP‐9 with the numbers of neutrophils and NCF2 expression in a four‐variable analytic setting (Figure 3B), and with the number lymphocytes vs. NCF2 (Figure 3C). Unlike MMP‐7, MMP‐2, MMP‐3 and MMP‐9 levels were correlated with the number of neutrophils and lymphocytes as well as the survival status.

### 3.5 The COVID‐19 ‘cytokine storm’ dissected into interleukins, chemokines and growth factors

**FIGURE 4:**
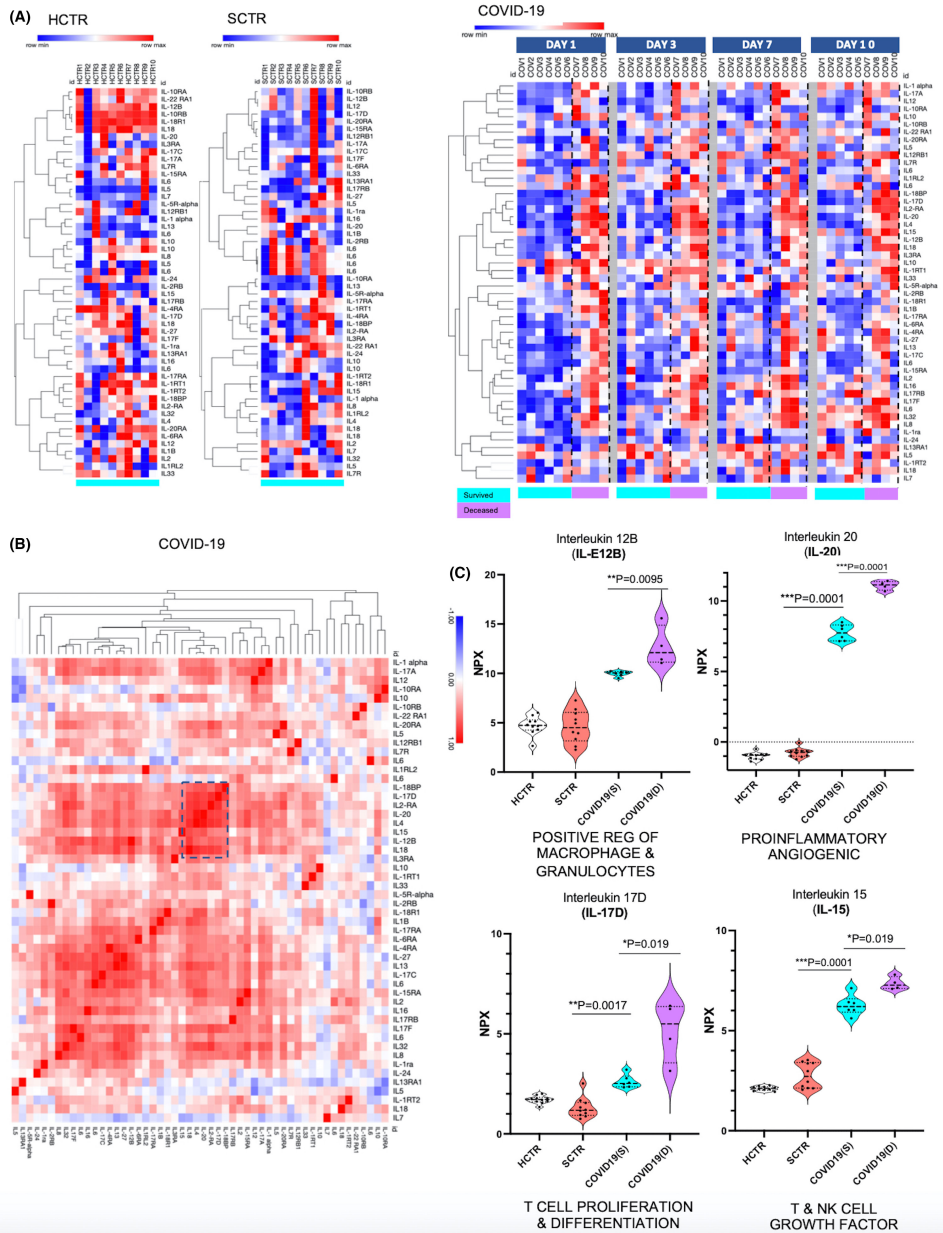
Interleukin role in the COVID‐19 ‘cytokine storm’ indicate immune cell specification, proliferation and systemic actions. (A) Heatmaps (left) represent interleukins signature of the control cohorts presented separately from their matched COVID‐19 counterparts (see Figure 3 for explanation). Heatmaps (right) refer to COVID‐19 interleukin signature and comprise the full set of interleukins from the Olink Explore 1196 library. Both maps were acquired by using the Morpheus tools repository as described in Figures 2A,3A. Conceptualization of data partition followed the same principles like in Figures 2, 3 (data segregation by survival and temporal means). (B) Similarity matrix shows the interrelation between the interleukins based on Pearson/Spearman correlation algorithms. The intense red indicates the highly correlated clusters. (C) The most significant interleukins that marked the ‘survival’ versus ‘fatal outcome’ in COVID‐19 patients were analysed as single markers (in violin plot format). IL‐12B indicates macrophage and granulocyte activity, IL‐20 has a vascular pro‐inflammatory role, IL‐17D is a T/NK‐cell growth factor, and IL‐15 a T‐cell differentiation trigger.

Interleukin (IL) levels are displayed in heatmaps where at least 14 were clustered in COVID‐19 patients with fatal outcome (Figure 4A). Among these are IL‐1α (a macrophage product that serves B‐cell maturation) and IL‐17 (A, D and F: NF‐kB‐controlled immune functions, including upregulation of other inflammatory interleukins such as IL‐6). We also confirmed IL‐4 and IL‐2RA in the COVID‐19 inflammatory environment.^14^ Other interleukins upregulated in plasma of deceased patients were IL‐18BP (a Th1 immunity responder involving IFNγ upregulation), IL‐15 (neutrophil activity and phagocytosis), IL‐20 (angiogenesis and EC communication with granulocytes governed by TGFB1 and NFkB), IL‐32 (T/NK‐cell activator and TNF inducer) and IL‐13R (an interleukin associated with asthma). In Figure 4B, the entire interleukin panel was analysed in a similarity‐map that demonstrated different degrees of correlation between different molecules. Based on correlation, several candidates for individual marker analysis were selected: IL‐20 (a pro‐inflammatory angiogenic), IL‐12B (a positive regulator of myeloid lines), IL‐17 (a supporter of T‐cell activity) and IL‐15 (a growth factor for T and NK cells). These latter interleukins were significantly higher in COVID‐19 patients with fatal outcome (Figure 4C).

**FIGURE 5:**
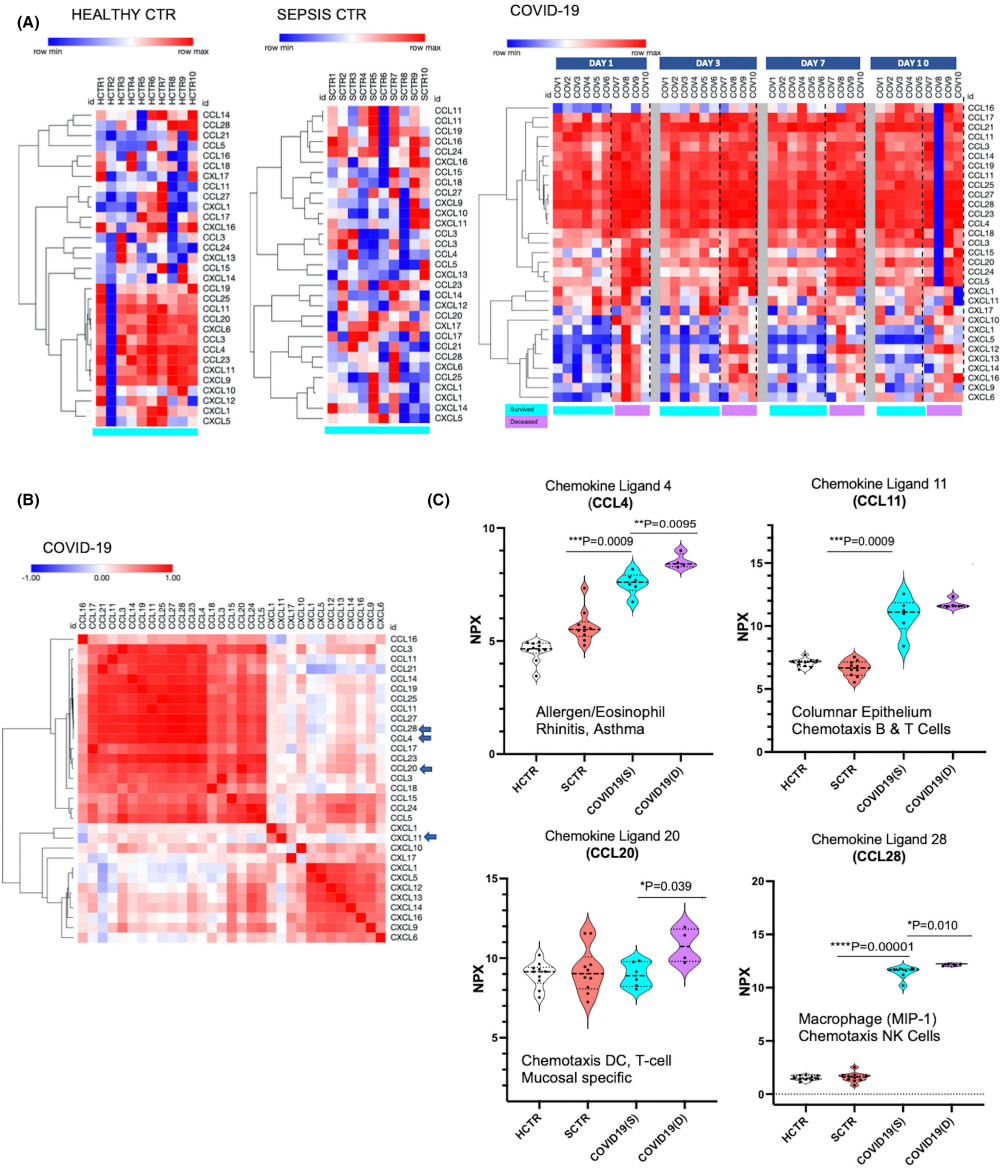
Chemokine role in the COVID‐19 ‘cytokine storm’ indicates a strong blast with critical ligands originating in the respiratory mucosa and the columnar epithelium. (A, B) Heatmaps representing chemokine signatures for COVID‐19 patients and controls (HCTR and SCTR) were processed in the same fashion and following the same principles explained in Figures 1A,3A and 4A. (C) Showcased (violin plots) are the chemokines that mark the respiratory tract‐specificity: CCL11—an allergen (with eosinophiles chemotaxis) particularly in asthma and allergic rhinitis; CCL20 mucosal‐specific dendritic cell (DC) chemotaxis acting on T cells; CCL4 columnar epithelium chemotaxis for T and B cells; CCL28/MIP‐1, a mucosal macrophage chemotaxis factor operating on the NK cells.

While interleukins could be compensatory and palliative to the destructive effect of the cytokine storm, plasma chemokine secretion demonstrated a persistent blast (Figure 5A, far right heatmaps). There were three distinct groups of chemokines: (1) common throughout all the severe COVID‐19 patients (CCL4, 11, 14, 16, 1719, 25, 27 28), (2) common but significantly increased in the patients with fatal outcome (CCL18, 3, 15, 20, 24, 5, CXCL1 and 11, CXL17), and (3) specific to the deceased patients (CXCL1, 5, 12, 13, 14, 16, 9, 6). CXCL1 has chemotactic activity for neutrophils and mediates inflammatory signals on endothelial cells in an autocrine fashion. Among the chemokines associated with a fatal outcome, functions are shared in a selective fashion for different types of cells as follows: CXCL5 (together with CXCR2 recruit neutrophils and promote angiogenesis with connective tissues remodelling), CXCL12 (a chemoattractant for T‐lymphocytes and monocytes, but not neutrophils) and CXCL14 (attracts neutrophils and a limited number of dendritic cells, but not T or B cells, monocytes, natural killer cells or granulocytes), CXCL16 (a scavenger receptor on macrophages) and CXCL9 (attracts T cells). Furthermore, the three groups of chemokines described above were highly correlated and formed three distinct clusters in a similarity‐matrix format (Figure 5B), which predicts functional interrelationships. From this analysis, Figure 5C showed four individual chemokines with significant change in COVID‐19 (CCL4, CCL‐11, CCL‐20 and CCL‐28), which could determine the immune status of the respiratory tract with two chemokines being mucosal (CCL20) and columnar epithelium (CCL11) specific.

**FIGURE 6:**
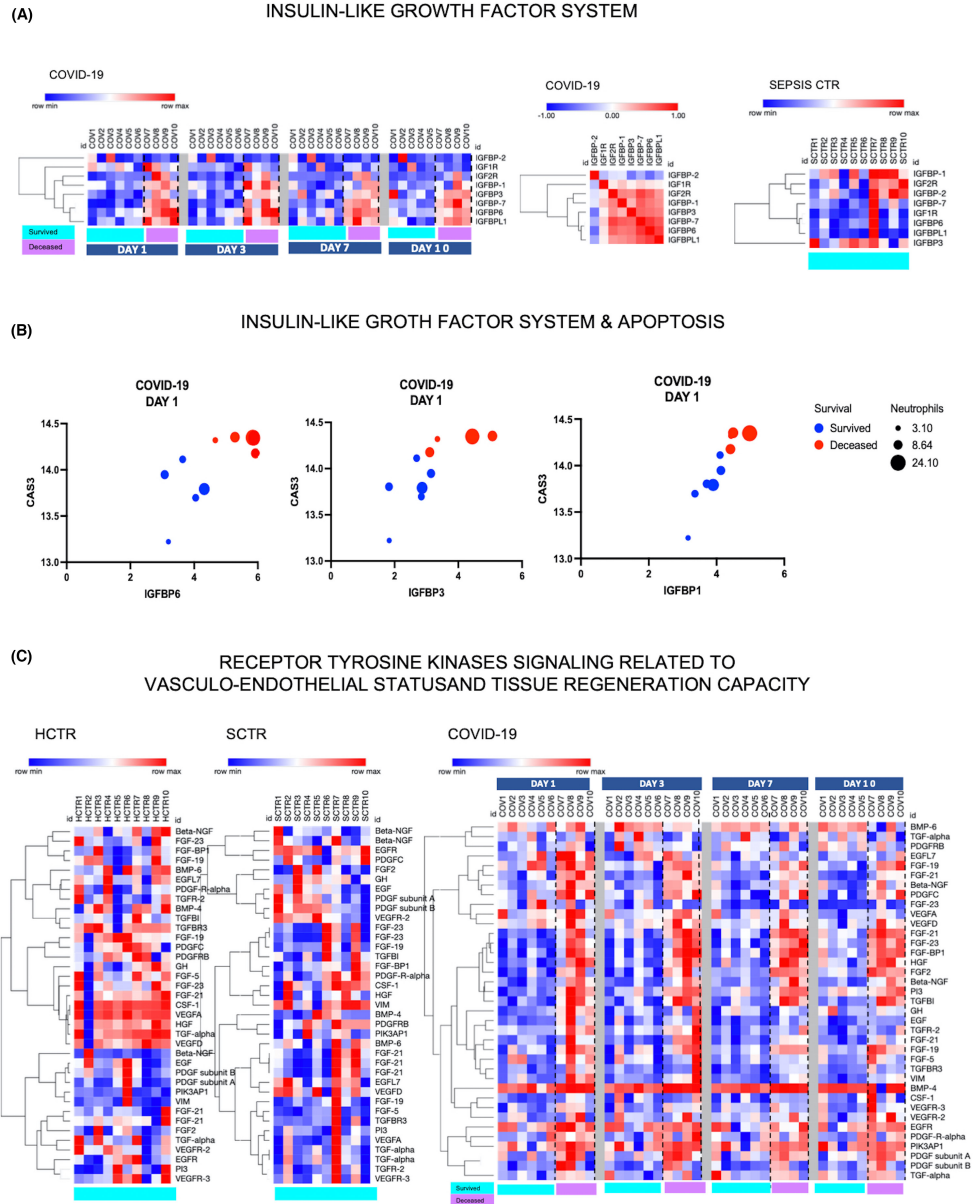
Growth factors (GF) with signalling through receptor tyrosine (RTK) are components of the ‘cytokine release syndrome’ in COVID‐19, with predicted alleviating‐regenerative role. (A) Clustered heatmap (a Morpheus software output) showing the presence of insulin‐like growth factor (IGF) system in the plasma. The IGF system consists of RTK receptors (IGF1R and IGF2R) and insulin‐like growth factor binding proteins (IGFBP‐1, IL‐2, IL‐3, IL‐6 and IL‐7) which are well represented in COVID‐19 plasma but not in SCTR. (B) IGFBP‐1, IGFBP‐3 and IGFBP‐6 are highly upregulated in COVID‐19 and correlated with the neutrophil numbers, NCF2 levels and survival. Graphs show the results of a multiple‐variable correlation analysis where *r* > 70% was considered significant (NCSS tools, 2021 version). (C) Heatmap visualization of growth factor signalling through receptor tyrosine kinases (RTK), together with their key partners

The third ‘cytokine storm’ compartment consisted of growth factors and their modulators (Figure 6), which are important for organ sustainability and regeneration. Group 1 was the insulin‐like growth factor (IGF) system and the afferent binding proteins (IGFBPs), presented in Figure 6A. Along with growth hormone (GH), both IGF‐I and IGF‐II were downregulated in COVID‐19 (Figure S1). IGFIR and IGFIIR were also downregulated; however, several IGFBPs were upregulated in deceased COVID‐19 patients (Figure 6A). Among these, IGFBP‐3 and IGFBP‐6 are known to be involved in apoptosis.^33^ IGFBPs also have IGF‐independent functions and some act intranuclearly, such as the IGFBP‐6 that is involved in DNA‐repair.^33^, ^34^, ^35^, ^36^, ^37^, ^38^ We analysed IGFBP‐1, IGFBP‐3 and IGFBP‐6 in relation to Caspase‐3, patient survival status and the numbers of neutrophils in a four‐variable format (Figure 6B), and observed that their actions were found to be correlated. Other growth factors that signal through receptor tyrosine kinases like IGFs were also downregulated in COVID‐19, including vascular endothelial growth factors (VEGF‐A, VEGF‐B and VEGF‐C), several fibroblast growth factor (FGF) isotypes and colony stimulating factor‐1 (CSF‐1). These latter growth factors show a low regenerative potential for the damaged tissues. The down‐regulation of the VEGFs suggests that the inflammation‐induced permeability of vascular beds may become chronic in severe COVID‐19. In addition, FGF‐2, FGF‐21 and FGF‐23, as well as platelet derived growth factors (PDGFs), were upregulated, thereby supporting a role for circulating fibrocytes.^39^ One component of the growth factor compartment is BMP4, which was highly upregulated in all patients with COVID‐19. BMP4, as a member of the transforming growth factor‐β1 (TGF‐β1) superfamily, functions in many developmental processes, including neurogenesis, vascular development and angiogenesis, as well as organ sustainability after injury (Figure 6C). Biomarkers from Figures 2, 3, 4, 5, 6 have been sampled for validation by immune‐arrays that confirmed the results of the OLINK targeted proteomics (Figure S1A). The immune‐array readings were also processed for hierarchical clustering and similarity analysis (Figure S1B).

### 3.6 Tissue‐specific toxicity and organ damage are highly dominant in severe COVID‐19

COVID‐19 patients exhibited disease worsening process (Figure S2A). Among the critical markers: (1) FOXO1 (a transcription factor which is the main target of insulin signalling and that regulates metabolic homeostasis in response to oxidative stress), (2) BANK1 (a B‐cell receptor induced by Ca^2+^ mobilization), (3) RASSF2 (apoptosis and cell cycle arrest promoter), (4) BAMBI (TGF‐β effector) and (5) EGFL7 (regulator of vascular tubulo‐genesis and promoter of angiogenesis and endothelial cell adhesion). A dramatic change was observed between ICU Days 3 and 7. These latter factors are not associated with specific tissue or organ, as they indicate a general alteration of immune‐homeostasis.

We next investigated the functional enrichment offered by the organ damage proteomic library (Figure S2A). Included in the top annotations were protein phosphorylation, blood vessel development and negative cell proliferation. The main signalling pathways were led by angiopoietin, VEGFR and PDGF. With a probability ranging from −7.5 to −6.5 (Log10P), focal adhesion kinase (FAK) and protein tyrosine phosphorylation (PTK) were the key mechanisms in COVID‐19. These mechanisms interfere with the CXCR4 pathway and PTK activity, which control various cellular processes including growth, differentiation, metabolism, and motility of immune and epithelial cells. For deeper functional analysis, we employed the ‘STRING’ Protein–Protein Interaction Networks platform that helped identify markers linked to important functions, such as pro‐inflammation governed by NFkB (with NCF2 regulating programming of neutrophil phenotype), cell adhesion and homing, vascular endothelial inflammation, kinase‐regulated cell adhesion and polarity, and finally apoptosis (Figure S2A).

The apoptosis phenomenon was investigated separately (Figure S2B), where levels of Caspases 2, 3 and 8 were significantly higher in all critically ill patients from ICU Day 1, and with FAS, TRAF and TRAIL markers upregulated in the deceased individuals.

### 3.7 Potential high‐precision, personalized therapeutics and drug reproposing opportunities derived from the COVID‐19 plasma proteome

Several key markers were searched within the DRUG_BANK data warehouse, thereby identifying several new therapeutic targets in COVID‐19 (Table S2). For example, the increased FES kinase in severe COVID‐19 can be addressed by repurposing Fostamatinib (a cancer drug) with the purpose of reducing organ damage via inhibition of TNF, IL‐8 and aggressive action of GM‐CSF. Fostamatinib is still under clinical trial (NCT04579393) and investigators proposed its use as a SYK‐kinase targeted therapy for the immunological complications of hospitalized patients with COVID‐19.^40^ The major role of MMPs is to modulate inflammatory and immune processes and they are targets for repurposed therapy with cancer drugs, as they either activate or inhibit a variety of chemokines and cytokines. Candidate MMP inhibitors, such as Marimastat and Andecaliximab, given with Leucovorin and Periostat/Doxicycline could slow organ damage and the cytokine storm associated with COVID‐19. Finally, Emricasan, a pan‐Caspase inhibitor drug could be used in COVID‐19 patients to decrease cell death.

### 3.8 Temporal plasma proteome analysis reveals COVID‐19 sequential pathology starting with epithelial damage at Day 1

COVID‐19 plasma profiles of each time point (ICU Days 1 to 10) were differentially analysed by volcano plot analysis^41^, ^42^, ^43^ against HCTRs (Figure S3A). The total numbers of differentially expressed proteins (DEPs) gradually increased in plasma from Day 1 (454) to Day 10 (706). Day 1 analysis is crucial as it identified Keratin‐19 (KRT‐19) as being highly upregulated in COVID‐19 plasma, suggesting that the structural integrity of lung epithelial cells is compromised early. KRT‐19 is a lung cancer‐specific marker,^44^, ^45^, ^46^, ^47^, ^48^ which can be potentially used as an early COVID‐19 diagnostic marker. In addition to KRT‐19, IFN‐γ, IL‐6 and monocyte chemotactic protein‐3 (MCP3) are also detected at high levels on ICU Day 1.

For deeper evaluation, we employed a Venn analysis, which partitioned the data into common and unique markers at each time point (Figure S3B). A total of 101 markers were common between ICU Days 1 and 10, and each time point had unique markers (two markers at Day 1, 27 at Day 3, 25 at Day 7 and 62 at Day 10). Analysed for annotated functional enrichment, the common markers were associated with key biological processes, including neutrophil degranulation, inflammation, ECM remodelling, cell adhesion, migration and response to oxygen species (Figure S2C), where FDR <1%. The main signalling pathways implicated include PI‐3/urokinase/haemostasis, apoptosis (driven by TRAIL and Caspases) and extracellular matrix organization [LOGp(−12)–(6)]. We further analysed the unique markers of ICU Days 3 and 7 using STRING interacting proteins network in Figure S2D, plotted as heatmaps. Although not numerous (<30), these markers interact and associate with critical functions. At Day 3, we identified T/B/NK delayed cell activation, impaired vascular barrier functions and coagulation, decreased adhesion to ECM by endothelial cells and lymphocytes, flawed myeloid clearance and deficient antigen presentation. At Day 7, we identified cell‐ECM interaction suppression, unreliable immune cell homing, and reduced cell growth and differentiation. Taken together, these findings suggest that the temporal evaluation stratifies the disease severity and may drive future management strategies.

## 4 DISCUSSION

In this report, we used a targeted‐proteomic analysis that emphasized the temporal changes in COVID‐19 plasma profile. We provide a comprehensive summary of the systemic response to SARS‐CoV‐2, which clearly outlined the disease severity and differential profile as compared to both age‐ and sex‐matched SCTR and HCTR. Over 200 proteins associated with COVID‐19 severity were grouped to dissect the ‘cytokine storm’. In addition, the proteome was deconvoluted by critical cell types of either immune, endothelial or stromal origin. Enrichment by cell type deconvolution enabled us to better infer immune cell function and cellular communication in severe COVID‐19. Numerous inflammatory mediators were associated with COVID‐19 patient death (IL‐6, IL‐8, CXCL10 and CCL‐2, CCL‐7, CCL‐8, CCL‐20).^14^, ^19^, ^20^, ^21^, ^22^, ^23^, ^24^, ^25^ Unique to our study, and as compared to others,^14^ were the chemokine blast, ECM remodelling and collagen turnover due to fibroblast activity. Our data also suggested altered coagulation, activated platelets, deficient T‐ and B‐cell homing, T‐cell exhaustion, deficient organ regeneration and kinases that can be ultimately manipulated by cancer repurposed dugs.

For the major COVID‐19 cell‐type signatures, we leveraged scRNA‐seq datasets^31^, ^32^ and deconvoluted their relative contribution to the COVID‐19 plasma proteome based on severity. Additionally, we identified a dramatic switch between ICU Day 3 and Day 7 driving organ damage. Tissue‐specific intracellular death signatures indicated severe apoptosis starting at Day 1 for patients that ultimately died. The high levels of CAS‐3, CAS‐8 and CAS‐12 were accompanied by additional cell death markers, such as FAS and TRAIL. Concomitant elevation of MMPs (2, 3, 9), collagen, laminin and integrins indicated neutrophil activity that may underlie ECM remodelling.

Cell type deconvolution of the plasma profiles indicated an increase in neutrophil phenotype with a reset in NCF2 expression, which was significantly decreased in fatal COVID‐19 patients. Depressed NCF2 suggested immature neutrophils, as reported previously with an scRNA study of blood cells and validated by flowcytometry.^44^ Altered NCF2 expression observed in our data set was also consistent with its altered expression in purified circulating neutrophils obtained from COVID‐19 patients.^45^ As NCF2 is necessary for NADPH oxidase, our data suggested that reprogrammed neutrophils in COVID‐19 have limited ability to produce ROS,^39^, ^40^, ^46^, ^47^, ^48^, ^49^, ^50^, ^51^, ^52^ perhaps increasing patient susceptibility to secondary bacterial infections.

Decreased CD4 naive, Tγ/δ, B naive, Treg and B‐plasma cells were also observed in our data set, consistent with the significantly decreased B and CD4+ T cells that were previously reported.^44^ Using CIBERSORT, we further deconvoluted the plasma proteomic profile and demonstrated that B‐cell memory cells, CD8 and T‐cell follicular helper cells were increased. The apparent discrepancy between our study and Silvin et al.^44^ was the CD8 population, which was explained by the sample source and the indirect plasma measurement versus direct analysis of the cells. Regardless, the dramatic increase in CAS3 that we identified in plasma should not be neglected when analysing cell types, as it might have been generated by the CD8 cells.

Building on these data, we propose a model of COVID‐19 pathophysiology with immune response based on protein expression that identified myeloid activity (especially neutrophils and macrophages) along with extracellular matrix remodelling, cell death and organ regeneration mechanisms. We propose that early myeloid activation translated into large numbers of reprogrammed neutrophils (potentially immature) with disabled NCF2 function that could indirectly trigger ‘lymphocyte exhaustion’.^28^, ^29^, ^30^

Neutrophil reprogramming can also be associated with activated extracellular matrix (ECM). We found that MMPs are candidate markers that may contribute to tissue remodelling and release of multiple proteins including collagen species.^34^, ^36^, ^37^, ^38^ For example, collagen type I induces platelet aggregation through Factor XII.^53^, ^54^ Moreover, polymerized type I collagen given by intramuscular injection (Fibroque^IMR^ drug) is able to downregulate the ‘cytokine storm’ and combat the ECM degradation by reducing the expression of IL‐1β, IL‐8, TNFα, IL‐17, and upregulate certain key adhesion molecules (ELAM‐1, VCAM‐1, ICAM‐1). ECM disintegration would release cells from the affected tissues,^55^ and a broken ECM may trigger not only aberrant T‐cell homing but also their ‘exhaustion’ followed by apoptosis.^14^, ^36^ In addition, impaired adhesion can also stimulate massive chemokine release in COVID‐19 plasma, stimulating cell migration which could ultimately be detrimental to tissue stability.^14^, ^36^, ^37^, ^38^, ^56^, ^57^, ^58^ In fatal COVID‐19 patients, we found an increased release of MMPs, caspases, chemokines and organ damage biomarkers, thus unable to contain the inflammatory immune responses and failing organ regeneration.

The SLAM biomarkers were also upregulated indicating activation and differentiation of a wide variety of immune cells.^28^, ^29^, ^30^ Usually, SLAMF molecules positively regulate NK cell functions,^4^, ^9^ IL‐2, IFN‐gamma and IL‐4 production by germinal centres T follicular helper. In the same environment, HLA‐E, a critical marker for NK cell activity COVID‐19 complications, was also detected. We confirmed that HLA‐E variants are risk factors for severe COVID‐19, as previously suggested.^59^

A range of biomarkers reflecting ECM remodelling and fibrosis were identified predicting the decline of the lung function.^37^, ^55^ Overactive fibroblasts (EPIC software output) are suspected of fibrillar collagens (type I, III, V collagens) production, which are not permeable to oxygen, unlike the networking collagens of the basement membrane (types IV and VIII).^36^ To manage this condition, we propose MMP inhibitors as potential therapeutic agents for severe COVID‐19 lung injury, including Aprotinin, a nonspecific protease inhibitor.^38^ MMP‐9 has been suggested as a critical marker for COVID‐19 screening,^34^ and we further have shown that elevated levels of MMP2 predict COVID‐19 fatal outcome.

## 5 CONCLUSION

**FIGURE 7:**
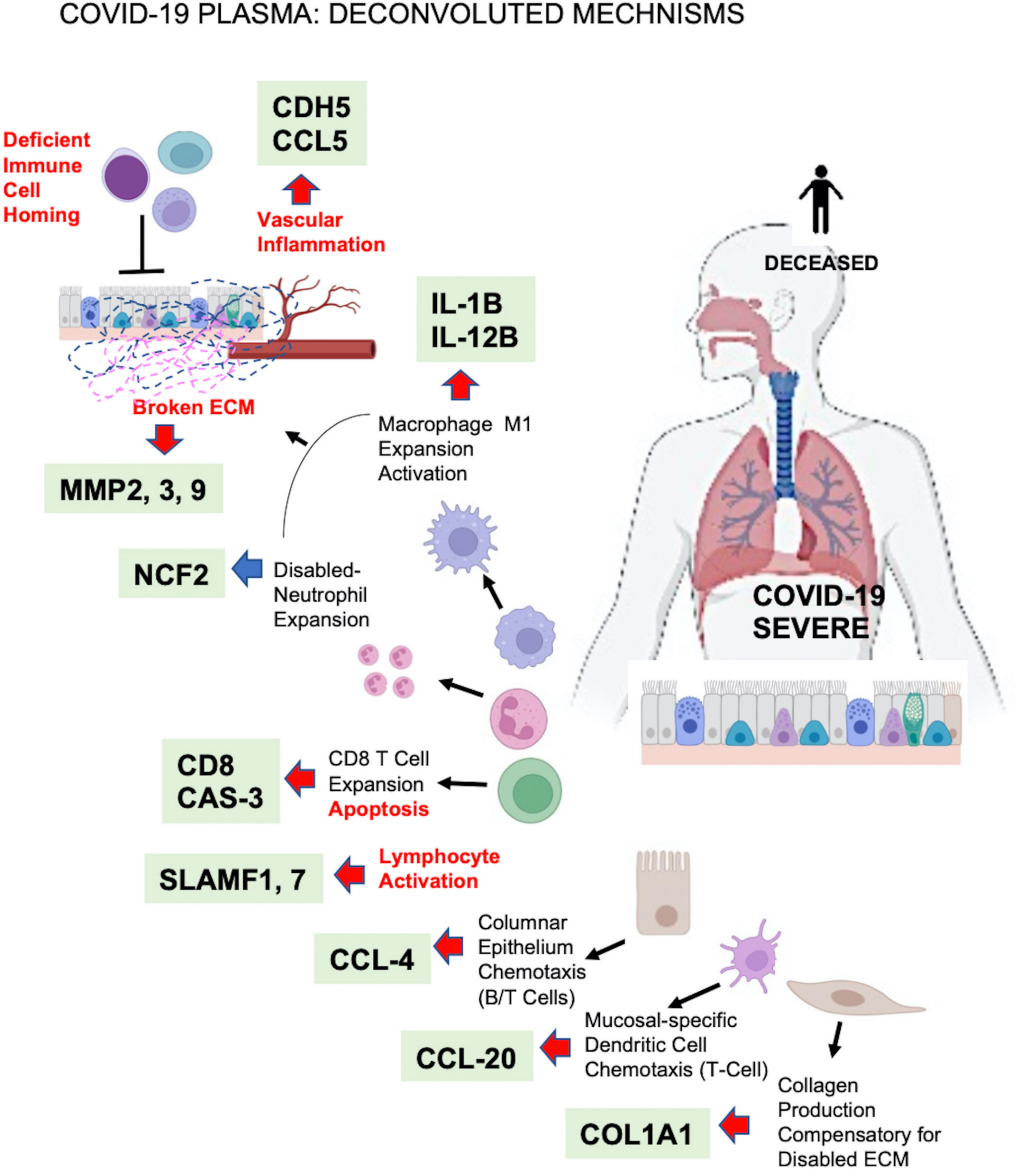
Summary cartoon illustrating the proposed deconvoluted mechanisms of COVID‐19 based on Olink analysis of the patient cohorts

Incorporation of the protein(s) into diagnostic schemes could stratify high‐risk patients for personalized therapies and interventional trials. Interestingly, several markers identified, such as the FES kinase, are regulated by cancer drug repurposing (Table S2). Our drug repurposing investigations suggested that cancer drugs, in particular, might alleviate certain pathologies in severe COVID‐19 that were due to proliferative processes mediated by growth factors acting through membrane receptor tyrosine kinases in the vascular bed.

(Figure 7), we propose that early activation of neutrophils and their reprogramming to low NCF2 release impairs myeloid activity, leading to (1) ECM remodelling; (2) release of collagen that activates platelets and induces coagulation; (3) deficient T‐ and B‐cell homing; (4) activation and expression of exhaustion markers on T cells; (5) cell death; and (6) deficient organ regeneration. Our model is not only consistent with the recently published reports^14^ but it brings further clarification and novelty with regard to severe COVID‐19 profiles. Finally, this study suggests future personalized diagnostics strategies to stratify high‐risk patients for personalized therapies and early disease management.

## AUTHOR CONTRIBUTIONS

**Cristiana Iosef:** Conceptualization (lead); data curation (lead); methodology (lead); software (lead); validation (equal); writing – original draft (lead); writing – review and editing (equal). **Claudio Martin:** Data curation (equal). **Marat Slessarev:** Data curation (equal). **Gediminas Cepinskas:** Methodology (supporting); writing – review and editing (supporting). **Carolina Gillio‐Meina:** Data curation (supporting); methodology (supporting); writing – review and editing (supporting). **Victor Han:** Investigation (supporting); visualization (supporting); writing – review and editing (supporting). **Douglas D. Fraser:** Conceptualization (lead); data curation (lead); formal analysis (lead); funding acquisition (lead); project administration (lead); resources (lead); supervision (lead); writing – original draft (lead).

## ACKNOWLEDGEMENTS

The authors are grateful to the frontline health professionals at London Health Science Center (London, ON, Canada) whom aided the collection of biological samples.

## FUNDING INFORMATION

This study was supported by the London Health Sciences Foundation and Academic Medical Organization of Southwestern Ontario (AMOSO) Innovation Fund (DDF, Principle Investigator).

## CONFLICT OF INTEREST

The authors state no conflicts of interest.

## Supporting information


**FIGURE S1:** **Validation of OLINK biomarkers by conventional immunoassay. (A)** Results of the OLINK data validation by immune‐arrays. **(B**) Brief correlation study by hierarchical clustering and similarity matrix. The experiment demonstrates that Covid‐19 plasma samples from a different patient pool than the one investigated by OLINK technology have the similar trend of biomarkers expression.

**FIGURE S2.** **COVID‐19 organ damage patterns**. **(A left)** Heatmaps emphasize COVID‐19 plasma profile on Days 1, 3, 7 and 10 comprised of the Olink‐1196 library targeting organ damage. The data were partitioned by temporal and survival criteria. The heatmap shows a clear change of pattern between ICU Days 3 and ‐7, consistent with patient deterioration patterns. **(A right top)** Mining this organ damage signature set we further inform on the functional enrichment and annotation of predicted Biological Processes (bar graph) and Signalling Pathways (table) using tools from the Gene Set Enrichment Analysis platform/repository (GSEA) at Broad Institute/Massachusetts Innovation and Technology (MIT, USA). The top pathways hits were around **i)** Focal Adhesion Kinase (FAK) regulated, ***ii)*** Protein‐Tyrosine Kinases (PTK) mediated, and ***iii)*** CXCR4 activity, which is consistent with this chemokine receptor mediating a large portfolio of inflammatory functions, adhesion and homing and hypoxia. **(A right bottom)** We further investigated the biomarkers that interact with each other directly or indirectly by using tools from the STRING platform (Protein–Protein Interaction Networks Functional Enrichment Analysis). The resulted biomarker selection is presented in a non‐clustered heatmap with functional specification for different groups of markers (GraphPad 9 output). Markers were grouped under proinflammatory functions with a reprogrammed/repurposed neutrophil phenotype (low in NCF2/ROS) followed by vascular irritation, kinases mediatory activity for cell adhesion and polarity gears, and finally apoptosis. **(B)** To complete the organ damage profile, a classical ‘apoptosis’ signature set was composed including biomarkers that cover the both triggers and effectors of programmed cell death, but not members of the necrotic pathways. Caspases (CAS3, 1 and 6) are highly present in the COVID‐19 and correlated with an abundance of TNF‐family markers, which indicate both organ damage and intense macrophage activity.

**FIGURE S3.** **Temporal partition of the COVID‐19 plasma proteome with identification of unique biomarkers for each time point that define the sequence of the disease progression. (A)** Volcano plots represent comparisons between proteomic data sets from COVID‐19 plasma Days 1 to 10 versus HCTR. The Y axis counts for the LOG10 q‐value, and the X‐axis for the difference between each COVID‐19 data point and the corresponding HCTR value. The number of the differentially expressed protein (DEP) markers grows from Day 1 (454) through to Day 10 (706), indicating a disease progression phenotype. **(B)** DEP of Days 1, 3, 7 and 10 have been Venn analysed and the common marker pool (101 proteins) was further investigated for functional enrichment (using tools from Gene Set Enrichment Analysis platform/repository (GSEA). **(C)** Signalling pathways for the common DEP pool are presented. COVID‐19 blood coagulation events are comprised under the ‘Haemostasis’ pathway. Other pathways are regulated by Urokinase and PI‐kinase that are likely regulating the thromboembolic disease (‘clot busting’ effect). Furthermore, apoptosis events regulated by Caspases and TRAIL are also predicted, along with significant extracellular matrix reorganization events. (D) Temporal evaluation of the unique interacting DEPs at ICU Day 3 and Day 7 show distinct profiles. After analysing protein–protein interaction/networking capacity (using STRINGdb repository tools), the interacting biomarkers have been plotted as heatmaps, where proteins were grouped and annotated for functional enrichment (GSEA functional assessment).

Table S1

Table S2

## Data Availability

Data available on request from the authors
