# Supplementary figures and images for: COVID‐19 plasma proteome reveals novel temporal and cell‐specific signatures for disease severity and high‐precision disease management
Source: J Cell Mol Med. 2022 Dec 19;27(1):141–57. doi: 10.1111/jcmm.17622 (PMC9806290; doi:10.1111/jcmm.17622)

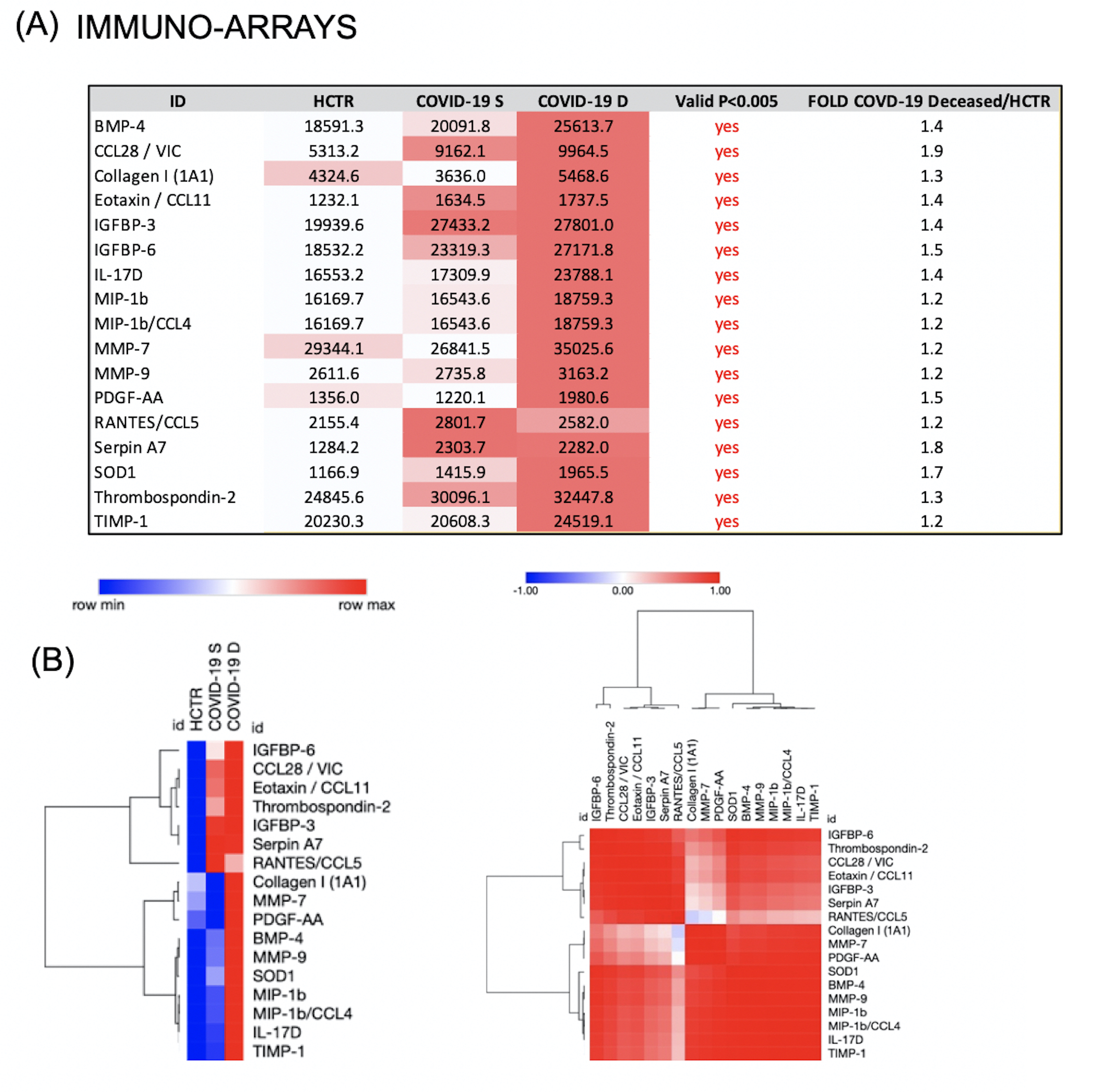

Supplement: Supplementary file 1 — FIGURE S1: Validation of OLINK biomarkers by conventional immunoassay. (A) Results of the OLINK data validation by immune‐arrays. (B) Brief correlation study by hierarchical clustering and similarity matrix. The experiment demonstrates that Covid‐19 plasma samples from a different patient pool than the one investigated by OLINK technology have the similar trend of biomarkers expression. [file JCMM-27-141-s001.jpg]

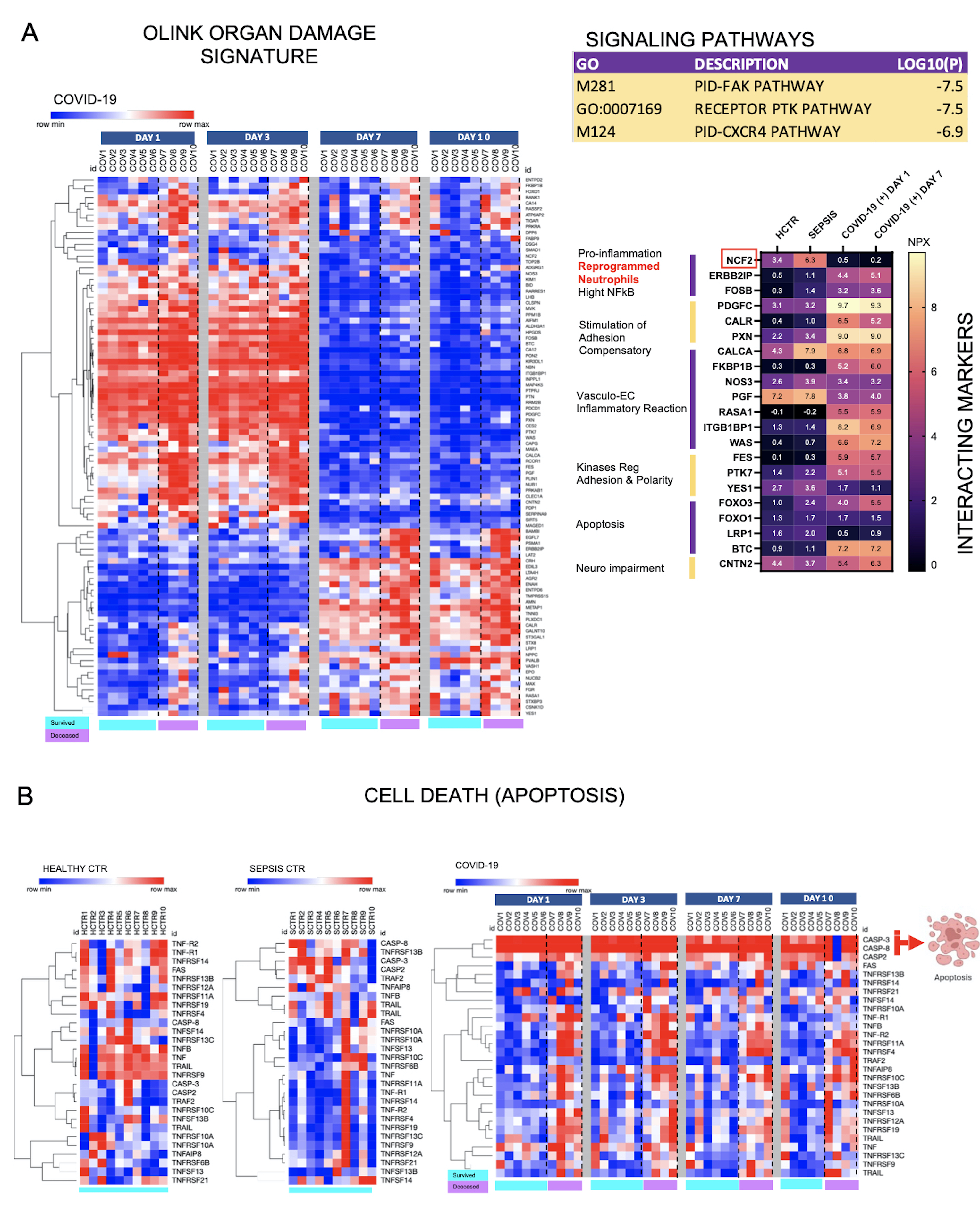

Supplement: Supplementary file 2 — FIGURE S2. COVID‐19 organ damage patterns. (A left) Heatmaps emphasize COVID‐19 plasma profile on Days 1, 3, 7 and 10 comprised of the Olink‐1196 library targeting organ damage. The data were partitioned by temporal and survival criteria. The heatmap shows a clear change of pattern between ICU Days 3 and ‐7, consistent with patient deterioration patterns. (A right top) Mining this organ damage signature set we further inform on the functional enrichment and annotation of predicted Biological Processes (bar graph) and Signalling Pathways (table) using tools from the Gene Set Enrichment Analysis platform/repository (GSEA) at Broad Institute/Massachusetts Innovation and Technology (MIT, USA). The top pathways hits were around i) Focal Adhesion Kinase (FAK) regulated, ii) Protein‐Tyrosine Kinases (PTK) mediated, and iii) CXCR4 activity, which is consistent with this chemokine receptor mediating a large portfolio of inflammatory functions, adhesion and homing and hypoxia. (A right bottom) We further investigated the biomarkers that interact with each other directly or indirectly by using tools from the STRING platform (Protein–Protein Interaction Networks Functional Enrichment Analysis). The resulted biomarker selection is presented in a non‐clustered heatmap with functional specification for different groups of markers (GraphPad 9 output). Markers were grouped under proinflammatory functions with a reprogrammed/repurposed neutrophil phenotype (low in NCF2/ROS) followed by vascular irritation, kinases mediatory activity for cell adhesion and polarity gears, and finally apoptosis. (B) To complete the organ damage profile, a classical ‘apoptosis’ signature set was composed including biomarkers that cover the both triggers and effectors of programmed cell death, but not members of the necrotic pathways. Caspases (CAS3, 1 and 6) are highly present in the COVID‐19 and correlated with an abundance of TNF‐family markers, which indicate both organ damage and intense macrophage a [file JCMM-27-141-s005.jpg]

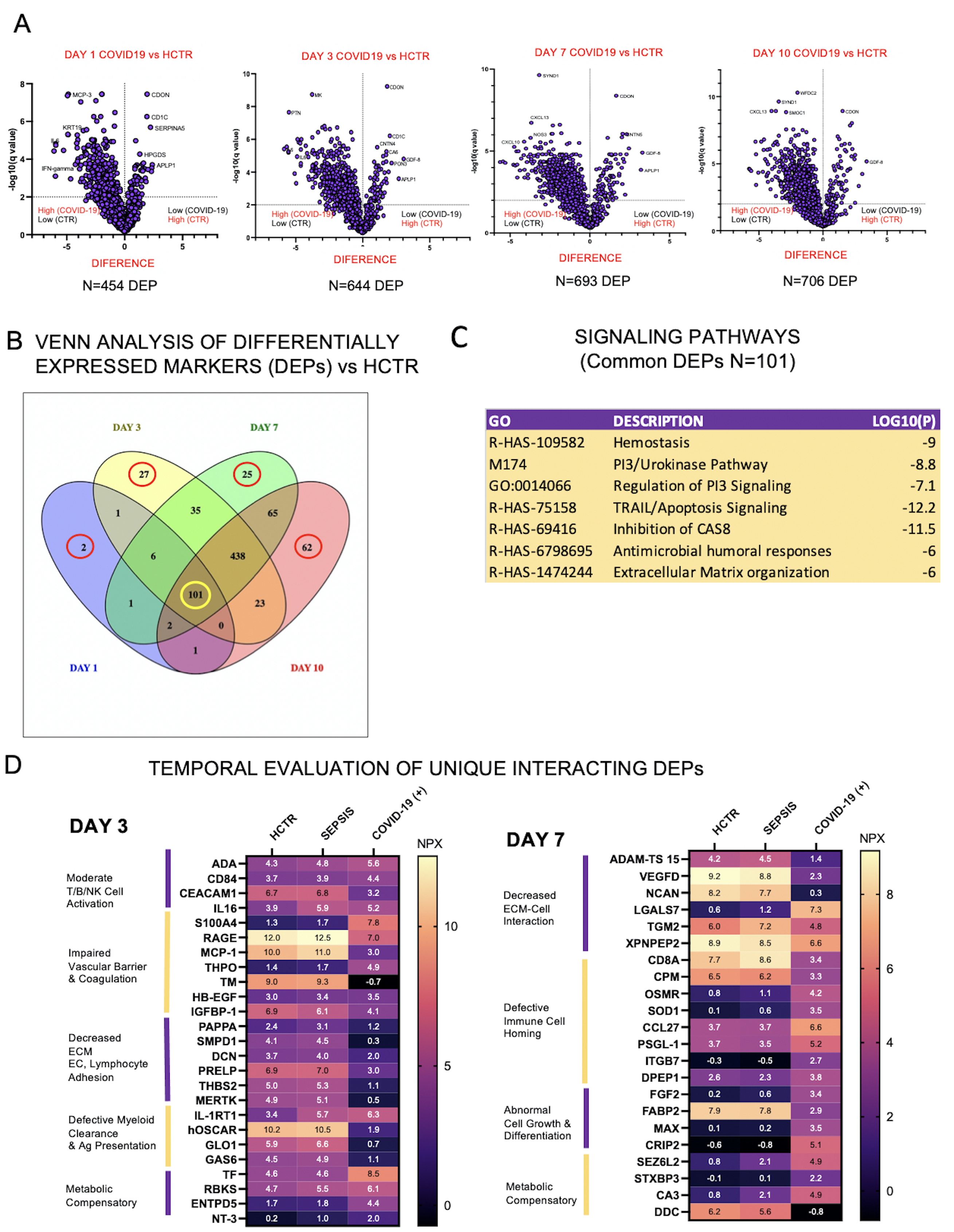

Supplement: Supplementary file 3 — FIGURE S3. Temporal partition of the COVID‐19 plasma proteome with identification of unique biomarkers for each time point that define the sequence of the disease progression. (A) Volcano plots represent comparisons between proteomic data sets from COVID‐19 plasma Days 1 to 10 versus HCTR. The Y axis counts for the LOG10 q‐value, and the X‐axis for the difference between each COVID‐19 data point and the corresponding HCTR value. The number of the differentially expressed protein (DEP) markers grows from Day 1 (454) through to Day 10 (706), indicating a disease progression phenotype. (B) DEP of Days 1, 3, 7 and 10 have been Venn analysed and the common marker pool (101 proteins) was further investigated for functional enrichment (using tools from Gene Set Enrichment Analysis platform/repository (GSEA). (C) Signalling pathways for the common DEP pool are presented. COVID‐19 blood coagulation events are comprised under the ‘Haemostasis’ pathway. Other pathways are regulated by Urokinase and PI‐kinase that are likely regulating the thromboembolic disease (‘clot busting’ effect). Furthermore, apoptosis events regulated by Caspases and TRAIL are also predicted, along with significant extracellular matrix reorganization events. (D) Temporal evaluation of the unique interacting DEPs at ICU Day 3 and Day 7 show distinct profiles. After analysing protein–protein interaction/networking capacity (using STRINGdb repository tools), the interacting biomarkers have been plotted as heatmaps, where proteins were grouped and annotated for functional enrichment (GSEA functional assessment). [file JCMM-27-141-s002.jpg]

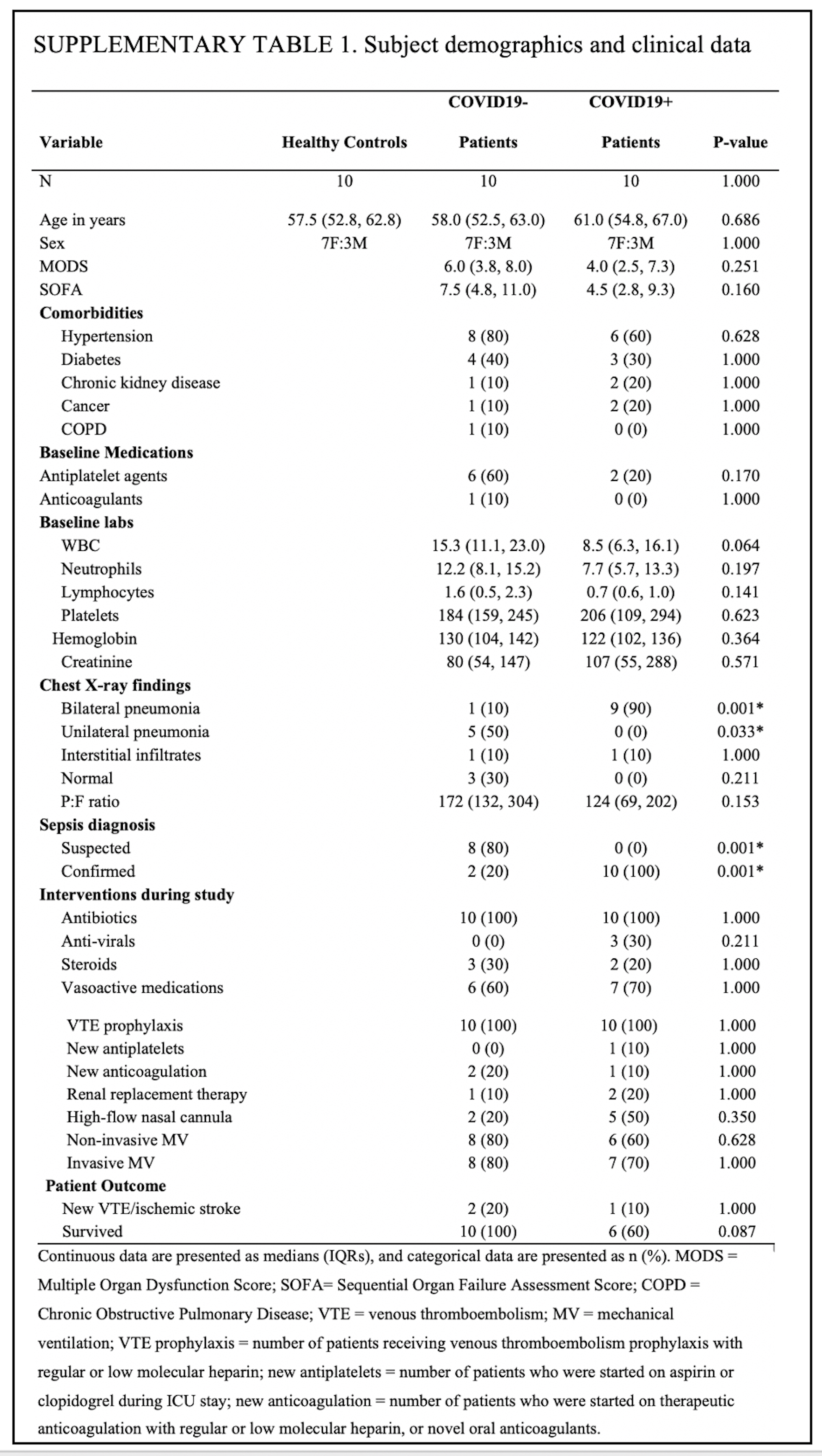

Supplement: Supplementary file 4 — Table S1 [file JCMM-27-141-s003.jpg]

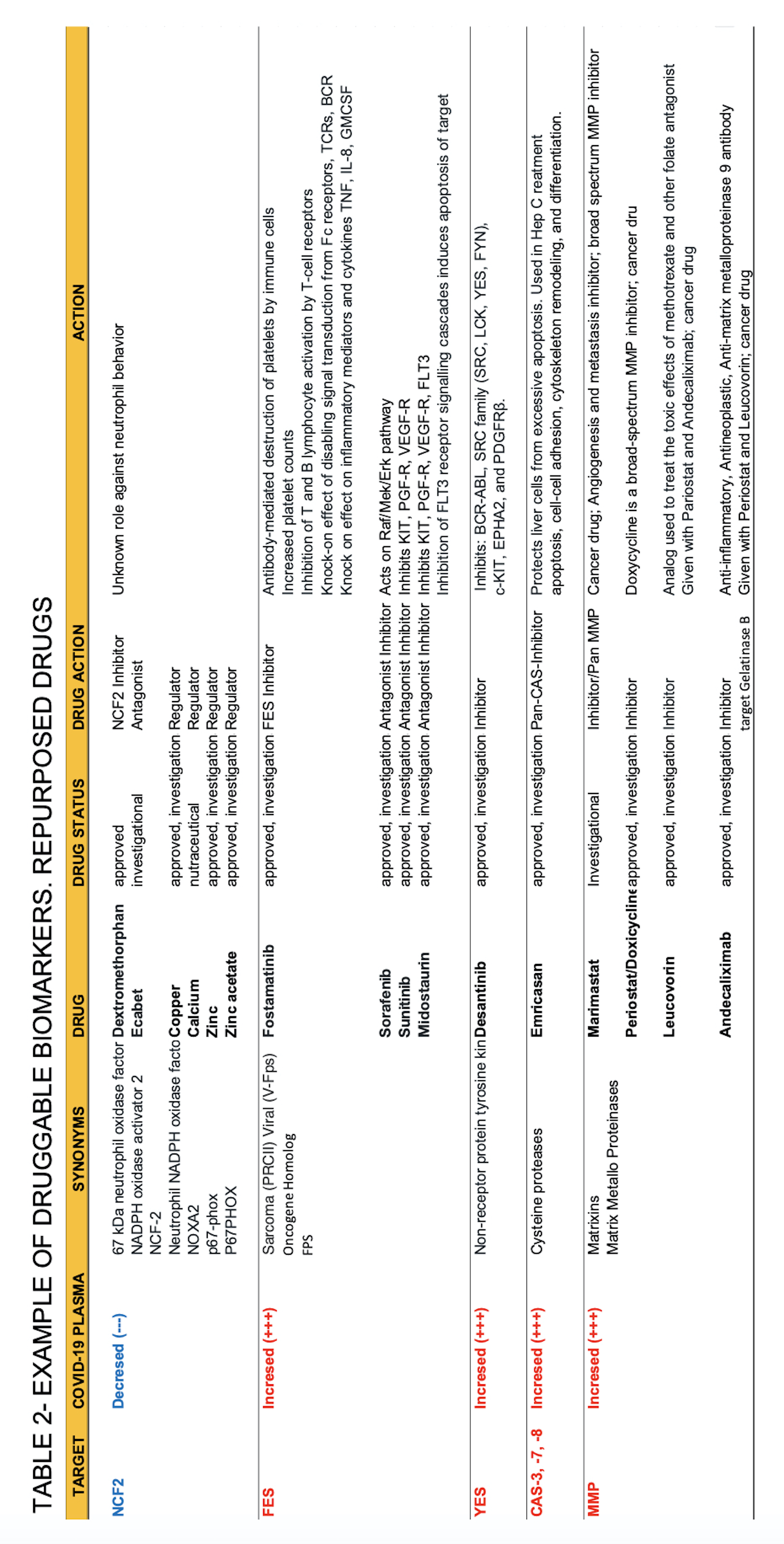

Supplement: Supplementary file 5 — Table S2 [file JCMM-27-141-s004.jpg]
